# The Cerebellum and Cognitive Function: Anatomical Evidence from a Transdiagnostic Sample

**DOI:** 10.1007/s12311-023-01645-y

**Published:** 2023-12-27

**Authors:** Indrit Bègue, Yannis Elandaloussi, Farnaz Delavari, Hengyi Cao, Alexandra Moussa-Tooks, Mathilde Roser, Pierrick Coupé, Marion Leboyer, Stefan Kaiser, Josselin Houenou, Roscoe Brady, Charles Laidi

**Affiliations:** 1 Department of Psychiatry, Beth Israel Deaconess Medical School & Harvard Medical School, Boston, MA USA; 2 Department of Psychiatry, McLean Hospital & Harvard Medical School, Boston, MA USA; 3 Department of Psychiatry, University Hospitals of Geneva & University of Geneva, Geneva, Switzerland; 4 INSERM U955, Institut Mondor de La Recherche Biomédicale (IRMB), Univ. Paris Est Créteil, Equipe 15 Neuropsychiatrie Translationnelle, Créteil, France; 5 La Fondation Fondamental, Créteil, France; 6 NeuroSpin, Neuroimaging Platform, CEA, UNIACT Lab, PsyBrain Team, Saclay, France; 7 Developmental Imaging and Psychopathology Laboratory, University of Geneva School of Medicine, Geneva, Switzerland; 8 Neuro-X Institute, École Polytechnique Fédérale de Lausanne, Geneva, Switzerland; 9 Institute of Behavioral Science, Feinstein Institutes for Medical Research, Manhasset, NY USA; 10 Division of Psychiatry Research, Zucker Hillside Hospital, Queens, NY USA; 11 Department of Psychiatry and Behavioral Sciences, Vanderbilt University Medical Center, Nashville, TN USA; 12 LABRI UMR 5800, CNRS, Univ. Bordeaux, Bordeaux INPTalence, France

**Keywords:** Cerebellum, Cognition, Cognitive flexibility, Healthy Brain Network, Structural neuroimaging

## Abstract

Multiple lines of evidence across human functional, lesion, and animal data point to a cerebellar role, in particular of crus I, crus II, and lobule VIIB, in cognitive function. However, a mapping of distinct facets of cognitive function to cerebellar structure is missing. We analyzed structural neuroimaging data from the Healthy Brain Network (HBN). Cerebellar parcellation was performed with a validated automated segmentation pipeline (CERES) and stringent visual quality check (*n* = 662 subjects retained from initial *n* = 1452). Canonical correlation analyses (CCA) examined regional gray matter volumetric (GMV) differences in association to cognitive function (quantified with NIH Toolbox Cognition domain, NIH-TB), accounting for psychopathology severity, age, sex, scan location, and intracranial volume. Multivariate CCA uncovered a significant correlation between two components entailing a latent cognitive canonical (NIH-TB subscales) and a brain canonical variate (cerebellar GMV and intracranial volume, ICV), surviving bootstrapping and permutation procedures. The components correspond to partly shared cerebellar-cognitive function relationship with a first map encompassing cognitive flexibility (*r* = 0.89), speed of processing (*r* = 0.65), and working memory (*r* = 0.52) associated with regional GMV in crus II (*r* = 0.57) and lobule X (*r* = 0.59) and a second map including the crus I (*r* = 0.49) and lobule VI (*r* = 0.49) associated with working memory (*r* = 0.51). We show evidence for a structural subspecialization of the cerebellum topography for cognitive function in a transdiagnostic sample.

**Supplementary Information:**

The online version contains supplementary material available at 10.1007/s12311-023-01645-y.

## Introduction

The cerebellum is a fascinating infratentorial brain structure with a pivotal role in human cognition [1]. Research on the cerebellum has been traditionally limited to its role in motor control, even though the majority of the cerebellar cortex is not involved in motor action planning or execution [2]. Cerebellar lesions across different diagnostic entities are associated with a diverse palette of cognitive deficits including disturbances of executive function such as planning, set-shifting, working memory, and verbal fluency [3].

A recent study linked cerebellar anatomy to cognitive functioning and found that anatomical features predicted both general cognitive function and psychopathology [4]. However, regional cerebellar morphometry differences relating to general cognitive function in psychosis [5] or in autism [6] were no different from controls. One reason for the discrepancy could be the difference in these studies’ approaches (dimensional vs. case–control comparisons). These studies looked into general cognitive function which may fail to capture anatomy-cognition links in the cerebellum because the relationships possibly concern specific cognitive domains. Studies are starting to progressively elucidate the functional organization of the cerebellum [2, 7–9]. Yet, a finer-grained nuanced investigation of the distinct facets of cognition is currently lacking leaving open the question of whether a structural cerebellar subspecialization exists with respect to cognitive abilities. Previous evidence showed that brain lesions in crus I and crus II, VIIB [10–12], and to a lower extent VIIIA and VI are associated with executive function performance. A seminal study in patients with cerebellar degeneration showed that distinct components of cognitive function (e.g., executive function, working memory, perceptual processing, and so on) relate differently to cerebellar topography [10]. Nevertheless, even though lesion studies are informative they have limitations, and a large-scale examination of the cerebellar mapping of distinct cognitive components is lacking. Furthermore, it is worth noting that psychopathology severity (e.g., levels of anxiety, depression, and so on) has not been systematically accounted for in the reviewed studies examining associations with cognitive function. Psychopathology severity impacts cognitive function [13] but also brain structure properties particularly in the developing brain [14–16]. Recently, cerebellar structure has been shown to be linked to both general cognitive function and psychopathology [4]. However, how cognitive mapping in cerebellar anatomy is represented independently of psychopathology contributions is not fully elucidated. Such investigation is of key interest with respect to the ensuing potential for clinical (e.g., neuromodulation) translation [17]. In sum, a major gap remains in the current understanding of cerebellar contributions to cognitive function and psychopathology: it is not clear whether subspecializations in cerebellar anatomy pertaining to components of distinct cognitive function exist and whether such differences can be observed independently of psychopathology severity.

In the current investigation, we examined for the first time how cerebellar regional anatomy may support cognitive function, capitalizing on a large dataset of transdiagnostic population employing a dimensional approach in agreement with the Research Domain Criteria framework [18]. Our aim was to first outline gray matter volume interindividual variability in the cerebellum across distinct facets of cognitive function (e.g., executive function, working memory, cognitive flexibility, processing speed). Considering we were interested in cohorts with detailed cognitive phenotyping, we used the Healthy Brain Network (HBN) [19], a landmark transdiagnostic mental health neuroimaging and behavioral dataset in a few thousand children and adolescents. This protocol was approved by the Chesapeake Institutional Review Board, is conducted following the Declaration of Helsinki for human research, and is described elsewhere [19]. The HBN includes predominantly unmedicated children and teenagers allowing us to examine cognitive cerebellar correlates unconfounded by chronic psychotropic consumption. Because spurious results can arise from quality control issues regarding neuroimaging scans, a rigorous quality assessment with visual inspection of all images is key to ensure the robustness of the results [6]. We used a data-driven multivariate canonical correlation analysis model (CCA) to evaluate the association between cerebellar anatomy and cognitive phenotype. Importantly, we used both permutation testing and bootstrapping to assess the significance and the robustness of our results.

## Methods and Materials

### Subjects

In the current investigation, we used data coming from an openly shared dataset, the Healthy Brain Network (HBN) project [19]. The HBN is a transdiagnostic dataset of neuroimaging and psychopathological assessments from a cohort of psychiatric or at-risk population of children and adolescents (5–21 years) [19]. Participants with severe neurological disorder or acute psychotic episodes are excluded in this cohort. In our study, considering our focus was on neurocognitive functioning, we excluded subjects with an intellectual deficiency (age-corrected IQ below 70), as measured with the Wechsler Adult Intelligence Scale (WASI-II) or the Wechsler Intelligence Scale for Children (WISC-V) [20]. The full clinical assessment of the HBN cohort is described elsewhere in depth [19].

### Assessments

We were motivated to examine distinct contributions of the specific cognitive function aspects to the cerebellar anatomy. In the HBN database, cognition is quantified via the NIH Toolbox Cognition domain [21]. The four subscales of the NIH Toolbox Cognition domain used in the HBN are detailed as follows. (1) The NIH Flanker assesses inhibitory executive control and attention and requires participants to focus on a target stimulus and ignore flanking stimuli. (2) NIH Card Sort assesses cognitive flexibility and requires participants to apply one rule to two target pictures (e.g., matching by color) and then another (e.g., matching by shape). (3) NIH List assesses working memory function and requires participants to sequence visually and orally presented stimuli, e.g., by size. (4) NIH Pattern Comparison Processing Speed Test assesses processing speed by requiring participants to compare two side-by-side pictures (same vs. different). In all subscales, higher scores mean better ability. We used the standardized normative scores for each subscale. Psychopathology severity is quantified with the Child Behavior Checklist (CBCL) [22], a widely employed scale that measures emotional, behavioral, and social problems in children and teenagers of 1.5–18 years old. These scales have a mean *t*-score of 50 with a standard deviation of 10. A *t*-score ≤ 64 indicates non-clinical symptoms, a *t*-score between 65 and 69 indicates problems rated high enough to be of concern but not overtly deviant, and a *t*-score ≥ 70 indicates clinical symptoms [22, 23]. Our selection of cognitive measures from the NIH Toolbox and the CBCL aimed to capture a relatively comprehensive range of cerebellum-associated cognitive domains [24–27], which are not only theoretically relevant but also practical for clinical assessment [28] and potential translational applications.

### MRI Acquisition

Acquisition of MRI scans was done in three sites in New York City: Staten Island, Rutgers University, and Cornell Brain Imaging Center. The specific details of each acquisition protocol are as follows: Staten Island images were acquired on a 1.5 T Siemens Avanto (*TR* = 2730 ms, *TE* = 1.64 ms, flip angle = 7°, slice number = 176, voxel dimensions = 1.0 × 1.0 × 1.0 mm^3^). Rutgers University images were acquired on a 3 T Siemens Tim Trio (*TR* = 2500 ms, *TE* = 3.15 ms, flip angle = 8°, slice number = 224, voxel dimensions = 0.8 × 0.8 × 0.8 mm^3^). Cornell Brain Imaging Center images were acquired on a Siemens Prisma 3 T MRI (*TR* = 2500 ms, *TE* = 3.15 ms, flip angle = 8°, slice number = 224, voxel dimensions = 0.8 × 0.8 × 0.8 mm^3^).

### MRI Processing

All subjects were processed using the CERES pipeline [29]. This fully automated method relies on a multi-atlas patch-based strategy that has been compared with manual tracing and performs well compared to other segmentation methods [30]. All structural T1 MRIs were processed by PC on a high computing performance cluster in Bordeaux, France. The CERES pipeline follows the parcellation protocol described in Park et al. [31], which provides a parcellation of the cerebellum and gray matter volumes for all cerebellar lobules except the cerebellar vermis, which is included in every lobule. Moreover, the CERES pipeline provides a mask of intracranial volume (ICV) [32].

### Quality Control

The quality control procedure was done in two steps: (1) visual inspection of the raw T1 images and (2) visual inspection of the images issued from the parcellation procedure in every slice for each spatial plan of the cerebellum by an expert rater (YE)—blind to the clinical features of each participant. We identified subjects with non-cerebellar voxels labeled as voxels belonging to the cerebellum, and vice versa, and subjects with parcellation errors within the cerebellar lobules. The same procedure has been applied previously [6]. No images with parcellation defects were included in further analyses. After the preprocessing and quality control step, we excluded 602 individuals after visual inspection of the raw T1 images (280 individuals), parcellation errors (279 individuals), low IQ (43 individuals), and incomplete psychometric scores (60 individuals). A final sample of 662 individuals was included in the subsequent neuroimaging analyses. A summary of the repartition of the excluded subjects can be found in supplementary figure S1.

### Statistical Analyses

#### Canonical Correlation Analysis

We performed our multivariate analyses with scikit-learn library [33]. We employed a regularized kernel canonical correlation analysis (CCA), using an open-source python *pyrcca* package [34], as a multivariate approach to evaluate the association between cerebellar anatomy (component with anatomical features “X”) and cognitive phenotype (component with clinical scores of interest “Y”). We performed *Z*-scoring on both the behavioral and anatomical matrices of our CCA model. We employed a linear regression model as implemented in the scikit-learn [35] library to control for scan location, age, and sex, calculating the residuals for subsequent use in our CCA model. Given evidence of a potentially complex relationship between ICV and cerebellar volume [36], we included ICV as a variable in our CCA to fully capture its association with cerebellar morphology.

In brief, CCA solves the canonical spaces in which the maximal correlation of projected datasets occurs, not pre-assuming the directionality of the relationship between datasets [37]. One documented disadvantage of CCA is overfitting to noise correlation of the datasets. To overcome this limitation, we implemented the algorithm used by the *pyrcca* toolbox. This algorithm constrains the number of components and find the optimal regularization parameters in a data-driven manner using a tenfold cross validation approach [34]. Here, we investigated a range of components ([2, 3, 4]) and a range of parameters ([0.0001, 0.01, 1, 100]). Such steps resulted in the best number of components of 2 and best regularization parameter of 0.0001 that were used for the subsequent analyses.

In our analysis, the anatomical component included our regions of interest, namely, the anterior lobe (lobules I–V), lobule VI, crus I, crus II, lobule VIIB, VIIIA, IX and X, and the ICV, after regressing out the effect of scan location, age, and sex. We included in the cognitive component the standardized scores of the subscales of NIH Toolbox, namely, List subscale indexing working memory, Card subscale indexing cognitive flexibility, Flanker’s subscale indexing cognitive control, and Processing subscale indexing processing speed. Multicollinearity was assessed with the variance inflation factor (VIF); values of VIF > 5 were considered to show multicollinearity [38] (see supplementary material 3). We then computed the correlation between the two canonical components for clinical and anatomical features. We repeated these analyses by including psychopathology severity quantified by the total *t*-score ofCBCL [28].

#### Assessment of Statistical Significance and Model Stability

Effect sizes were assessed with Cohen’s *d* and Pearson’s *r* correlation unless otherwise specified, following common statistical guidelines where Pearson’s *r* value around 0.1 is considered small, 0.3 moderate, and 0.5 large [39]. Regarding Cohen’s *d*, effect sizes were categorized as small (0.2), medium (0.5), and large (0.8) [39].

To assess statistical significance of our CCA model, we used non-parametric permutation testing [40]. Permutation testing involves random rearrangement of samples without replacement to estimate the population distribution and in turn, test the null hypothesis. Thus, *p* value would be defined as the proportion of permuted samples that test statistically higher than our observed sample. In this study, unless specified otherwise, the threshold of significance was set to *p* = 0.05 corresponding to the *r* value higher than the *r* value of the 95 percentile in a 10,000 random permutation test.

Crucially, we assessed the model stability through bootstrapping analysis [41]. Bootstrapping is used to create a sampling distribution by repeatedly taking random samples with replacement from the original sample. We then performed CCA on each bootstrapped sample and collected these results to perform summary statistics (mean and confidence intervals). The average estimated from these multiple random samples may be used to infer results regarding the robustness of the CCA results for the original sample. Here, we used a bootstrap of 10,000 random samples with replacement which showed a normal distribution of canonical correlations. The results were considered stable if the 95% confidence interval of the bootstrap distribution would not include zero. We assessed the effect of bootstrap rotation (supplementary material 4) and computed the bootstrap ratio (supplementary material 5) .

## Results

### Study Sample: Demographics and Psychopathology

**Fig. 1:**
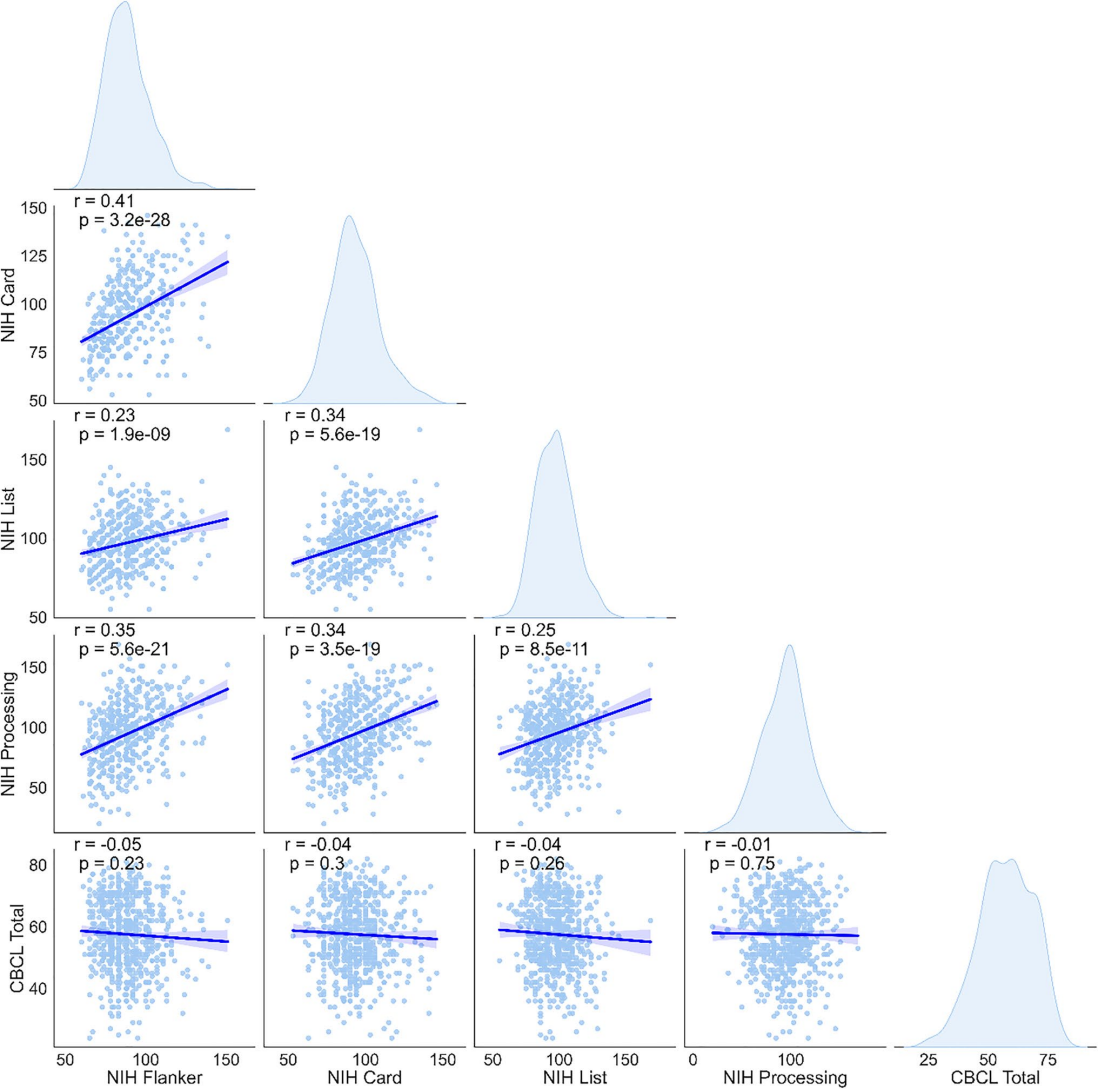
Scatterplot representation of the pair-wise correlation between psychopathology and cognitive characteristics (NIH Toolbox subscales) scores. Histograms of the distribution of each variable with smooth curves obtained using a kernel density estimate function. Abbreviations: NIH Toolbox, NIH-TB; NIH List, NIH TB List Sorting Working Memory Test; NIH Card, NIH TB Cognition Domain Dimensional Change Card Sort Test; NIH Flanker, NIH TB Flanker Inhibitory Control and Attention Test; NIH Processing, NIH-TB Pattern Comparison Processing Speed Test; CBCL total, total *t*-score of Child Behavior Checklist

The demographic characteristics of our sample can be found in Table 1 and supplementary material 1. In sum, the cohort included in our study (*n* = 662) had a mean age of 10.5 years [range 5.82–17.74 years old] and were predominantly males (58%). Next, we investigated the relationship between cognitive function assessed with the NIH-TB subscales and psychopathology severity quantified by the CBCL total *t*-score. The goal of this exploratory analysis was to assess the potential influence of psychopathological severity on cognitive measures, which could be a confounding factor in interpreting neuroimaging results. We focused on effect magnitude (defined by the *r* value), and since no significant correlations were found, multiple testing corrections were not deemed necessary. We found positive correlations between the individual cognitive subscales scores with small effect sizes (Fig. 1), indicating that these subscales measure little overlapping constructs. However, there were no associations of the cognitive subscales with psychopathology severity (CBCL) indicating no significant impact of psychopathology on distinct components of cognitive function.

**Table 1:** Summary of cohort characteristics (age, sex, scan site, and intracranial volume) and cognitive characteristics (NIH Toolbox subscales) of our cohort (*n* = 662). Smooth curves obtained using a kernel density estimate function. Abbreviations: *ICV*, intracranial volume; *NIH Toolbox*, NIH-TB; *NIH List*, NIH TB List Sorting Working Memory Test; *NIH Card*, NIH TB Cognition Domain Dimensional Change Card Sort Test; *NIH Flanker*, NIH TB Flanker Inhibitory Control and Attention Test; *NIH Processing*, NIH-TB Pattern Comparison Processing Speed Test; *CBCL total*, total *t*-score of Child Behavior Checklist; *CBIC*, Cornell Brain Imaging Center; *SI*, Staten Island; *RU*, Rutgers University; ‘*M*’, male; ‘*F*’, female

Variable	Count	Mean	Standard deviation	Min	Max
Age (in years)	662	10.5	2.91	5.82	17.74
Sex	{‘M’: 385, ‘F’: 277}				
ICV (in cm^3^)	662	1410.59	136.57	1027	1867.81
Scanning site: CBIC	285				
Scanning site: RU	315				
Scanning site: SI	62				
NIH Card	662	93.46	15.49	53	146
NIH Flanker	662	88.61	14.01	60	151
NIH List	662	97.13	14.69	55	169
NIH Processing	662	94.59	23.56	20	169
CBCL total	662	57.54	11.38	24.0	82.0

### Cerebellar Correlates of Cognitive Function

**Fig. 2:**
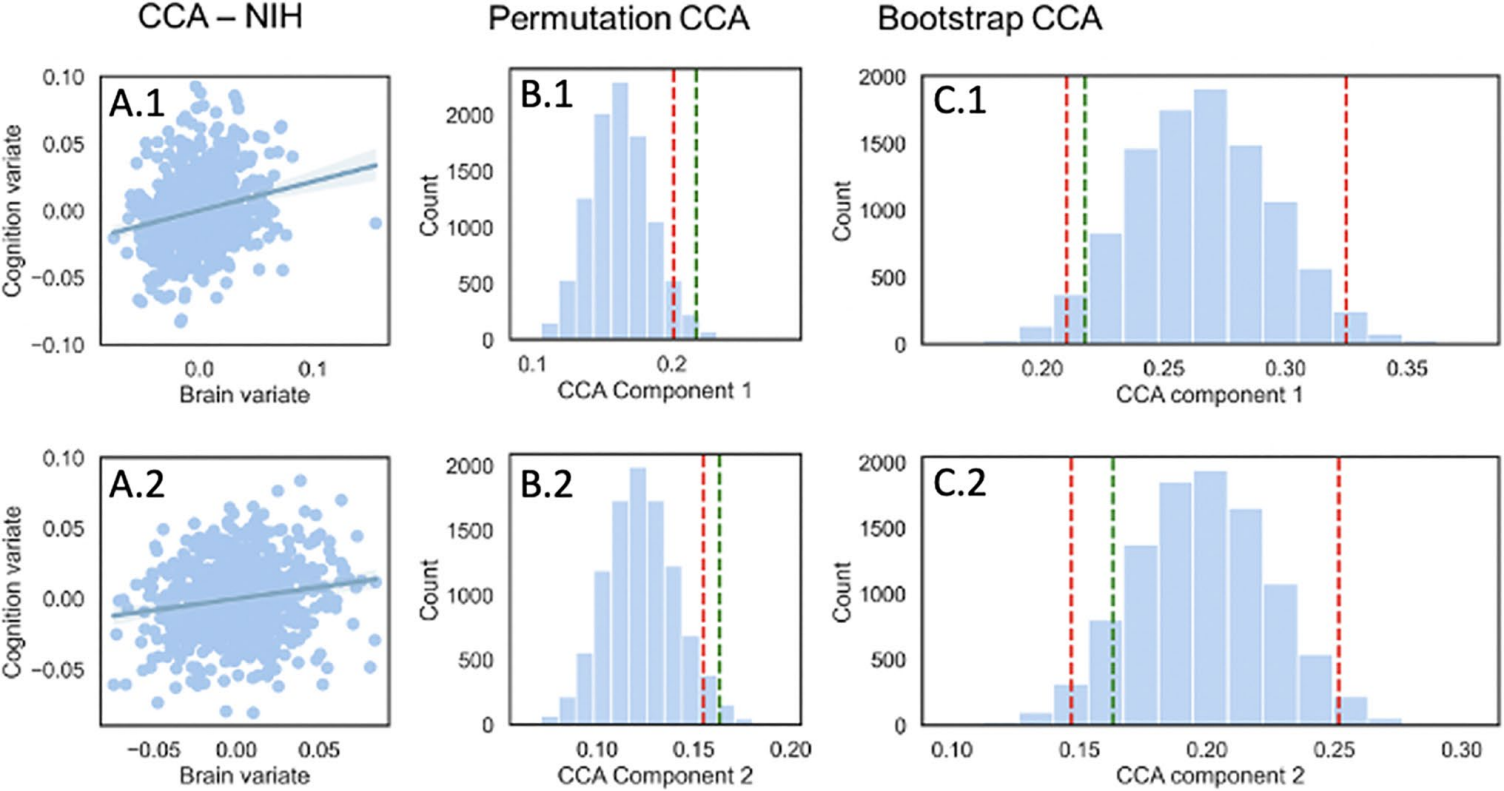
Canonical correlation analyses. A.1–2 Canonical correlation plots between the cerebellar and clinical variates (1: first pair of canonical variables, 2: second pair of canonical variables). B.1–B.2 Significance testing of the CCA. Distribution of CCA coefficients for component 1 (B.1) and component 2 (B.2) obtained by performing 10,000 permutations. Red line represents a significance threshold set for an alpha level of 0.05 (1: first pair of canonical variables, 2: second pair of canonical variables). C.1–C.2 Stability testing of the CCA. Distribution of canonical correlation coefficients between cerebellar and clinical variates by bootstrapping procedure with 10,000 tests at an alpha level of 0.05 for component 1 (C.1) and component 2 (C.2). Lower and upper bound corresponding to ± 1.96 SD in red dotted line (1: first pair of canonical variables, 2: second pair of canonical variables)

We then turned to the brain to examine whether we could uncover latent neural and behavioral dimensions to our data with a multivariate CCA approach (including age, sex, scan location, total intracranial volume, cognition (NIH-TB subscales)). Such an approach allowed us to identify two significant correlation between the first cognitive canonical variate (the subscales of the NIH Toolbox Cognition domain) and the first brain canonical variate (regional cerebellar gray matter volume and intracranial volume) at *r* = 0.22 with confidence interval [0.210–0.327], as well as between the second clinical canonical variate (the subscales of the NIH Toolbox Cognition domain) and the second brain canonical variate (regional cerebellar gray matter volume and intracranial volume) at *r* = 0.16 with confidence interval [0.148–0.254]. To assess significance, we conducted permutation testing with 10,000 tests at 95% that revealed that the correlation of both components was significant (statistical threshold for component 1: *r* = 0.20, and for component 2: *r* = 0.15) (Fig. 2).

Next, we performed a bootstrapping analysis to examine the stability of our multivariate CCA model [42]. Our results showed that our CCA results are stable and non-zero within the [5:95] confidence interval of the results generated by bootstrapping analysis.

**Fig. 3:**
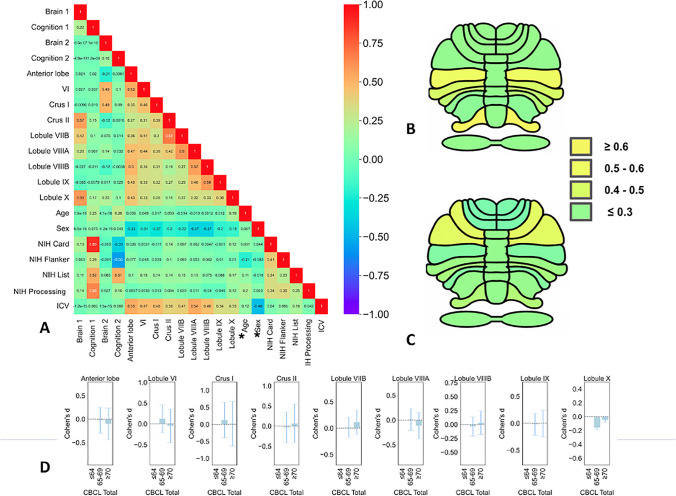
Relationship of cerebellar structure with cognition and psychopathology. **A**–**C** Canonical correlation analysis: loading of cognitive and anatomical variates as well as the correlation between all variables included in the anatomical and behavioral latent variable, age and sex. *Age and sex were regressed out from the brain variable. **D** Effect sizes as standardized mean difference in groups stratified by psychopathology severity quantified by CBCL *t*-score. Annotations: Brain 1, first anatomical component; Brain 2, second anatomical component; Cognition 1, first cognitive component; Cognition 2, second cognitive component; NIH Toolbox, NIH-TB; NIH List, NIH TB List Sorting Working Memory Test; NIH Card, NIH TB Cognition Domain Dimensional Change Card Sort Test; NIH Flanker, NIH TB Flanker Inhibitory Control and Attention Test; NIH Processing, NIH-TB Pattern Comparison Processing Speed Test; ICV, intracranial volume; CBCL, Child Behavior Checklist total *t*-score

Regarding the first component, cognitive flexibility (indexed by the NIH Card subscale), processing speed (indexed by NIH Processing scale), and working memory (indexed by the NIH List subscale) loaded the most on the clinical canonical variate at 0.89 (large effect), 0.65, and 0.52 (moderate effects), respectively. Gray matter volume in the crus II and lobule X loaded with a moderate effect size on the brain canonical variate at 0.57 and 0.59, respectively (Fig. 3). Regarding the second component, working memory (indexed by the NIH List subscale) and cognitive control (indexed by NIH Flanker subscale) loaded with a moderate effect on the cognitive canonical variate at 0.51 and − 0.56, respectively. Gray matter volume in the crus I and lobule VI loaded also moderately on the brain canonical variate at 0.49 each.

We then asked whether the variability in cerebellar volumes observed with cognitive function could be explained by clinical psychopathology severity. The goal was to examine variability in cerebellar volumes using a stratification that aligns more readily with clinical practice and possibly allows groups with meaningful clinical relevance. To perform this, we stratified the total CBCL *t*-score in the established normative cut-offs of the CBCL with a *t*-score of ≤ 64 for non-clinical symptoms, a *t*-score between 65 and 69 for borderline individuals with risk for problem behaviors, and a *t*-score ≥ 70 for clinical symptoms [22, 23]. We then computed the effect size (Cohen’s *d*) of differences in cerebellar volume using standardized mean differences accounting for age, sex, scan location, and intracranial volume. We found no significant differences in cerebellar volumes across these categories (Fig. 3D) showing a lack of psychopathology effects in cerebellar anatomy. Furthermore, we included the CBCL total *t*-scores and the NIH subscales in the same CCA model with permutation and bootstrapping. This analysis did not impact our main cognitive-cerebellum CCA results remaining both statistically significant and stable (supplementary material, Figure S3). We then asked whether specific dimensions of psychopathology could drive cerebellar variability and performed CCA analyses with the subscales of the CBCL. These results were not statistically significant and did not survive bootstrapping (Supplementary material, Figure S4). In sum, our results indicated that cognitive function but not psychopathology severity drove the observed cerebellar anatomic variability.

## Discussion

Cognitive neuroscience is only beginning to unravel the role of the cerebellum in higher “supratentorial” cognitive functions. Despite historically being framed as a “motor control” brain region, extensive human neuroimaging and lesion evidence has suggested a cerebellar role in cognitive function. Furthermore, previous evidence uncovered a correspondence of cerebellar anatomy with general cognition and psychopathology. Therefore, we set out to examine the cerebellar topography in connection to specific components of cognition. Our multivariate analyses (CCA) outlined how different components of cognitive function map onto cerebellar morphometry independently of psychopathology severity in support of the cerebellar cognitive and affective syndrome [3, 43]. In particular, we showed only partly shared cerebellar maps of cognitive function (Fig. 3A–C): a first map encompassing cognitive flexibility (large effect size) and speed of processing (moderate effect size) associated with regional gray matter volume in crus II and lobule X and a second map including the crus I and lobule VI associated with cognitive control (moderate effect size). Working memory associations were similarly present in both these maps (crus II, lobule X, crus I, and lobule VI) with similar moderate effect sizes. These results account for psychopathology severity and other confounds and suggest that such correspondence between cerebellar anatomy may go across transdiagnostic boundaries. Crucially, permutation testing and bootstrapping analyses showed that these relationships are significant and our CCA model is robust and stable [42].

Our findings highlight an association of the cerebellar crus II and lobule X structure with cognitive flexibility with a large effect size. Importantly, we show that variability in cerebellar gray matter volume is linked to cognitive flexibility abilities in a dimensional across-diagnostic categories manner. While theoretical accounts have posited a role for the cerebellum in the flexible coordination of cognitive processes (Cognitive Dysmetria theory), strikingly, no large-scaled evidence for such contribution existed prior to our work. Animal research evidence indicated that hemi-cerebellectomized animals are unable to flexibly switch to a new set of rules, despite having intact motor responses [24, 44]. A few small-sample human studies [10, 45–52] have underlined a cerebellar role in mediating cognitive flexibility. The cerebellar correspondence of cognitive flexibility fits well with evidence from cerebellar lesions [11, 12, 16, 53] and theoretical accounts of cognitive dysmetria theory [54]. According to this theory, the cerebellum plays a key role in coordinating different cognitive and affective processes, quite similarly to its role in motor coordination. Impairments in cognitive flexibility seem to be prevalent in a variety of psychiatric disorders across the lifespan [55–59] and they represent a potentially important pharmacological [60], psychotherapeutic [61], or neuromodulation [62] target.

Our results point to two cognitive-anatomical maps both related to working memory in agreement with previous findings [8, 9, 63–66]. Furthermore, we show a first component characterizing the positive association of cognitive flexibility and speed of processing with regional gray matter volume in crus II and lobule X and a second, separate, component that captures the relationship of crus I and lobule VI with working memory. Speed of processing (the ability to quickly process information), working memory (the ability to hold and manipulate information during short periods of time), and cognitive flexibility (the ability to switch rapidly between mental states and tasks) are interconnected cognitive capacities that are important for flexible behavior. Previous literature has focused on supratentorial prefronto-striatal networks of working memory and cognitive flexibility [67–69]. In this circuitry, cognitive flexibility have been shown to rely on dopaminergic signaling in the striatum, as demonstrated in human PET neuroimaging [70–72] and task-related studies [73, 74], and in the dorsolateral prefrontal cortices [75]. In line with these findings, Westbrook and Braver [69] attributed a key role to dopaminergic neurons in the flexible coordination of cognitive processes for goal-directed behavior. In light of our results, we speculatively propose that, similarly, dopaminergic signaling may underlie cerebellar participation in cognitive flexibility or stability either through direct local dopaminergic signaling in the cerebellum or via indirect (e.g., distant) cerebellar prefrontal closed loops as part of “the rich club” [76]. Indeed, high levels of dopamine have been found in postmortem cerebellum of humans, rats, and monkeys reviewed in Flace et al. [77]. Further research is needed to examine whether and how dopaminergic signaling underlies cerebellar correlates of cognitive function.

Our study has several strengths. First, we overcome previous shortcomings of case–control studies by endorsing a dimensional approach that captures phenotypic gradients in a large cohort. To our knowledge, this is the largest study to date to ever examine cerebellar contributions to distinct cognitive components including cognitive flexibility. Second, we employ a validated pipeline (CERES) with careful and stringent quality control to ensure optimal preprocessing and avoid spurious results. In the current work, we have included only high-quality imaging data surviving a stringent visual quality check (e.g., only 662 of the images have passed the quality check of the initial *n* = 1452 subjects, Figure S1) using the same quality control protocol employed previously [6]. Third, this study provides statistically significant (permutation testing) and stable (bootstrapping) data-driven results in the largest transdiagnostic sample to date to ascertain the significance of the results and the stability of the CCA model. Permutation testing allowed us to establish that the obtained results are statistically significant. However, obtaining significant results does not exclude the possibility of having a random sampling error (e.g., the sample does not represent the general population). Such a possibility can be ruled out by bootstrapping: the results of the original cohort can be compared with bootstrapped samples, allowing us to examine the robustness of the model. Here, our results survived bootstrapping and we can confidently state that our results are not due to a random sampling error. Our study advances the field by revealing structural correlates of cognitive performance within the cerebellum in a large, transdiagnostic pediatric sample. We provide novel insights into the cerebellum’s contribution to cognitive flexibility, employing a rigorous quality control process and a structural MRI approach, with a dimensional perspective that aligns with the RDoC initiative [18]. This methodology allows for the identification of associations that transcend traditional diagnostic categories, potentially leading to more nuanced understanding and targeted interventions.

Regarding limitations, our cross-sectional design and correlational analyses prevent inferences on the causal nature of the observed interindividual variability in the cerebellum. Additionally, it is important to acknowledge that, while our cross-sectional study design offers valuable insights into transdiagnostic patterns among pediatric and adolescent populations, it inherently limits our capacity to capture the dynamic nature of cerebellar maturation [78] and its interaction with cognitive development over time. Future longitudinal studies are essential to fully delineate these developmental trajectories and their implications for cerebellar-behavior relationships. Moreover, the cognitive subscales used in this cohort are only a subsample of all cognitive function processes. In addition, these findings may not generalize to older populations considering that in this cohort only young developing individuals (mean age of 10.5 years) were included. Future follow-up studies should examine how these relationships are expressed in adult cohorts. Finally, our study does not allow us to directly examine the effects of diagnostic categories on cerebellar structure, due to the high rate of comorbidities in this transdiagnostic sample that made category-based analyses less fitting. We instead opted for a dimensional approach, utilizing the CBCL scale. One caveat of our study is the absence of a separate validation sample, which could further strengthen the reproducibility of our findings and should be the focus of future efforts. In addition, we focused on structural lobular parcellations of the cerebellum to enable strict QC. While applying a functional atlas to structural data could provide valuable insights, QC process is considerably more challenging with functional parcellations, particularly when ensuring the precision needed for valid interpretations of our results.

Our work links cerebellar morphometry to distinct components of cognitive function including cognitive flexibility. These functions are observed to be altered in psychiatric disorders such as schizophrenia, depression, autism, and obsessive–compulsive disorders [79], all of which also are shown to have cerebellar aberrations [80]. Given the recent advance in cerebellar non-invasive brain stimulation and its association with neuroimaging [17, 81], our work opens the perspective of cerebellar targeting across different psychiatric diagnoses for cognitive improvement. Overall, our results elucidate for the first time the cerebellar anatomical circuitry supporting interindividual differences in cognitive function and highlight a prominent role for the human cerebellum in distinct aspects of cognition for flexible adaptive behavior.

### Supplementary Information

Below is the link to the electronic supplementary material.

Supplementary file1 (DOCX 1.14 MB)

## Data Availability

Data are available on request from the authors.
