# Supplementary material for: The Cerebellum and Cognitive Function: Anatomical Evidence from a Transdiagnostic Sample
Source: Cerebellum. 2023 Dec 27;23(4):1399–410. doi: 10.1007/s12311-023-01645-y (PMC11269336; doi:10.1007/s12311-023-01645-y)
Supplement: Supplementary file 1 — Supplementary file1 (DOCX 1.14 MB) [file 12311_2023_1645_MOESM1_ESM.docx]

# Supplementary material

### Supplementary Material 1. Diagnostic categories in the study sample

|  | **Study Sample**  **(N =662)** |
| --- | --- |
| **Diagnosis**  ADHD  Anxiety Disorder  Specific Learning Disorder  ASD  Depressive Disorders  Communication Disorder  Motor Disorder  Other  No diagnosis | 275 (41.5%)  84 (12.7%)  57 (8.6%)  41 (6.2%)  40 (6.0%)  12 (1.8%)  3 (0.5%)  59 (8.9%)  91 (13.7%) |

### Supplementary Material 2. Linear univariate analyses

We also examined post-hoc linear univariate relationships of cerebellar regional gray matter to cognitive abilities measured by NIH toolbox subscales controlling for age, ICV, and psychopathology severity (indexed by CBCL total scores) as continuous variables and sex, scan location as categorical variables (Figure S2). We found a main positive effect of NIH List subscale indexing working memory in gray matter volumes of the anterior lobe (p=0. 015), Lobules VI (p = 0.0008), Crus I (p = 0.0008), Crus II (p =0.001), VIIB (p =0.0008), VIIIA (p=0.003) and X (p = 0.0008). Further, we found a main positive effect of NIH Card indexing cognitive flexibility in Crus II (p =.001), VIIB (p=.015), VIIIA (p=0.03) and Lobule X (p=.004). Cognitive control (indexed by NIH Flanker’s subscore) correlated with gray matter volume in the anterior lobe gray matter volume (p=0.04) and Crus II (p=0.03) whereas processing speed (indexed by NIH processing subscore) positively correlated to gray matter volume in Crus II (p=0.04) and Lobule X (p= 0.04) (all p-values are FDR corrected).

###

### Supplementary Material 3 : Assessment of the multicollinearity in behavioral variables with the variance inflation factor (VIF)

We did not find evidence of a strong multicollinearity between behavioral variables

| Scale | NIH Card | NIH Flanker | NIH List | NIH Processing | CBCL Total |
| --- | --- | --- | --- | --- | --- |
| VIF Score | 1.346 | 1.2914 | 1.1619 | 1.2263 | 1.003 |

Legend : VIF Score > 5 suggests a strong multicollinearity

### Supplementary Material 4 : Effect of potential axis rotation

To mitigate the potential impact of axis rotations and reflections in our CCA results, we employed a bootstrapping strategy with Procrustes correction (Krishnan et al. 2011). This technique was applied to each bootstrap resample to ensure correction for axis rotations and reflections. Each of the procrustes-adjusted bootstrap samples were then subjected to CCA to confirm the stability and reproducibility of the canonical variates across different iterations (Canonical Variate 1: mean correlation = 0.2655 confidence intervals [0.211 - 0.328], Canonical Variate 2: mean correlation = 0.20, confidence intervals [0.151- 0.255].


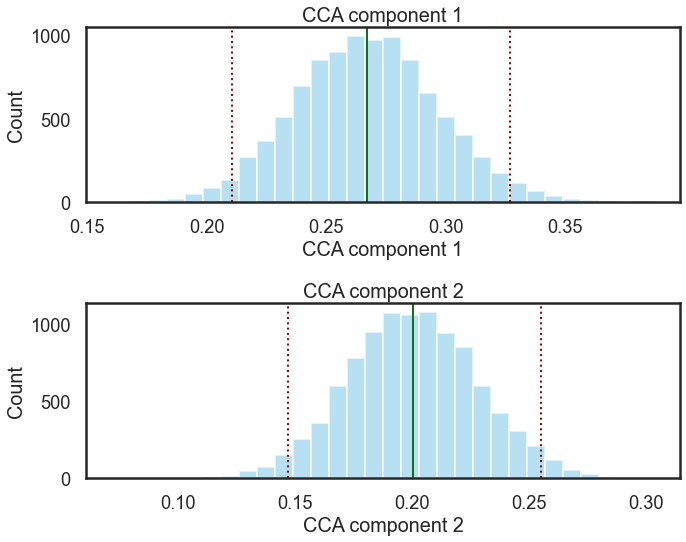


### Supplementary Material 5 : Bootstrap Ratios (BSR)

We report the Bootstrap Ratios (BSRs) for the first and second canonical components derived from our analysis. These BSRs were calculated from 1000 bootstrap samples by dividing the original correlations by their respective standard deviations in the boostrapped samples, providing a measure of significance relative to the bootstrap variability. The threshold for significance is set at BSR > 1.96 which determines whether the original correlation is statistically significant at a 95% confidence level.

We found that for CCA component 1, the BSR is 7.247, and for CCA component 2, the BSR is 6.324, meaning that the canonical correlations are statistically significant relative to the bootstrap variability.

### Supplementary Figures

###
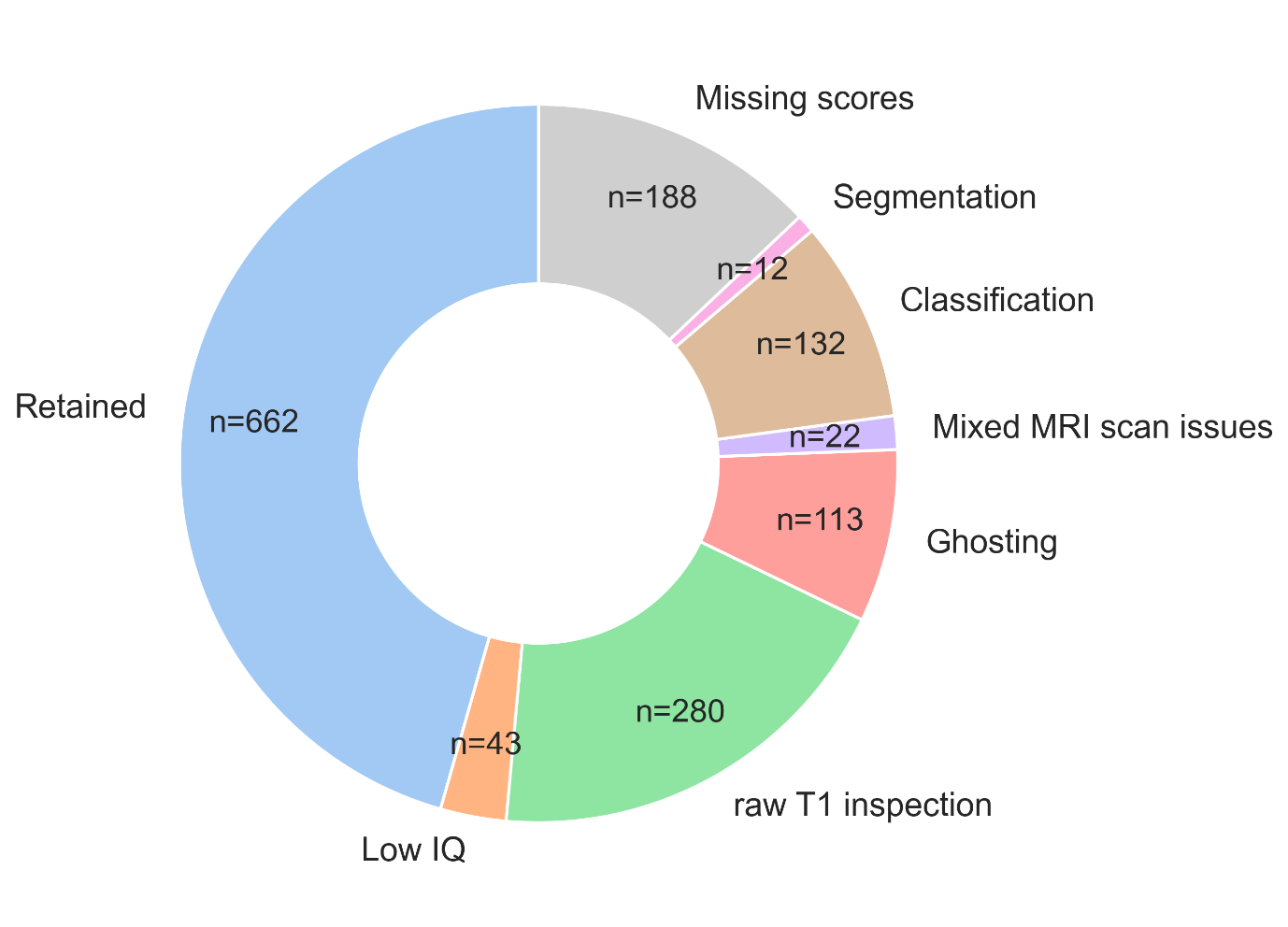


**Figure S1**. Repartition of excluded individuals from the final analysis and the respective percentages.


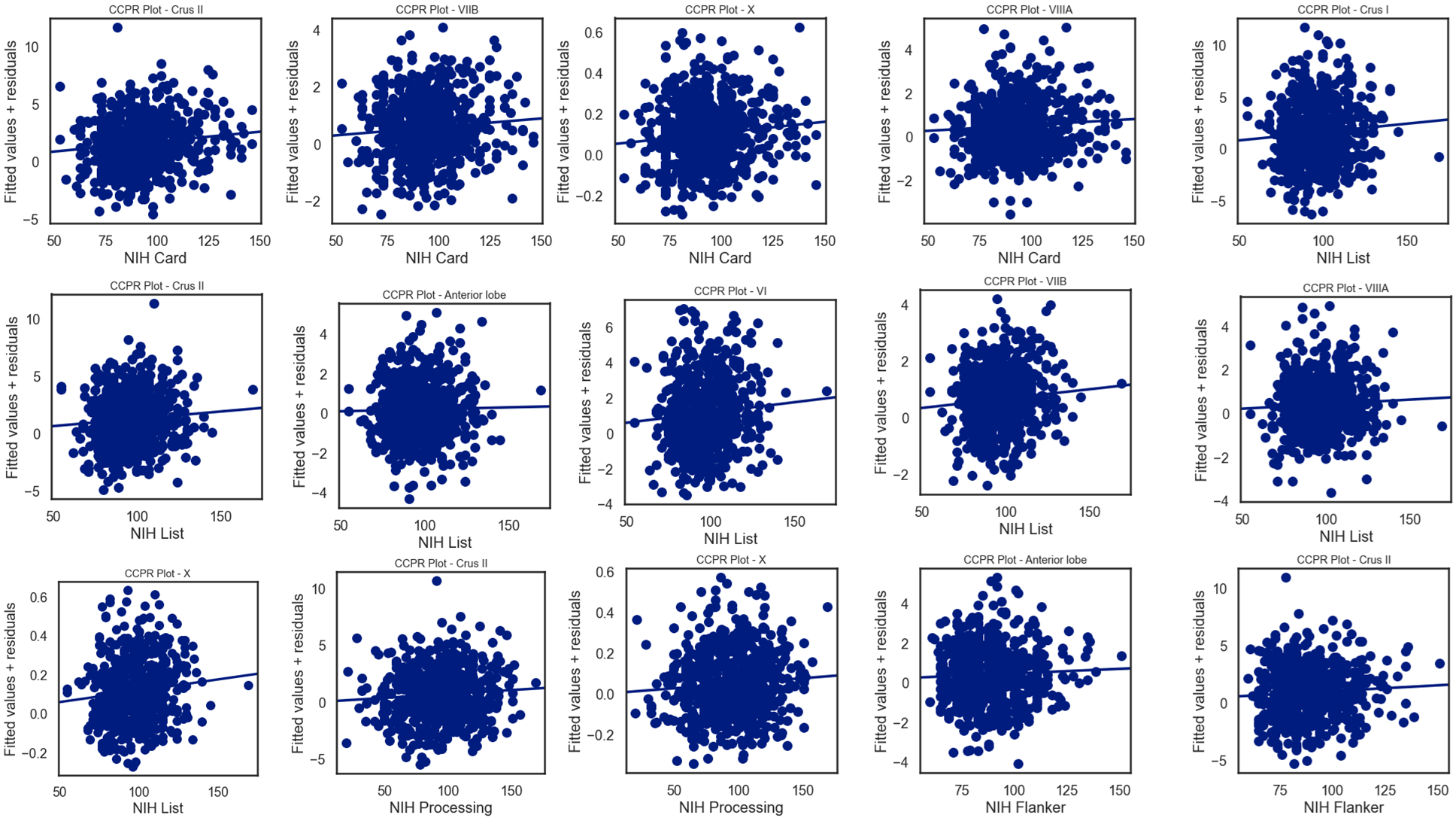


**Figure S2**. Summary of univariate analysis statistically significant results (p<0.05 FDR corrected). Component and component-residual (CCPR) plots of NIH subscales with cerebellar regional gray matter volume


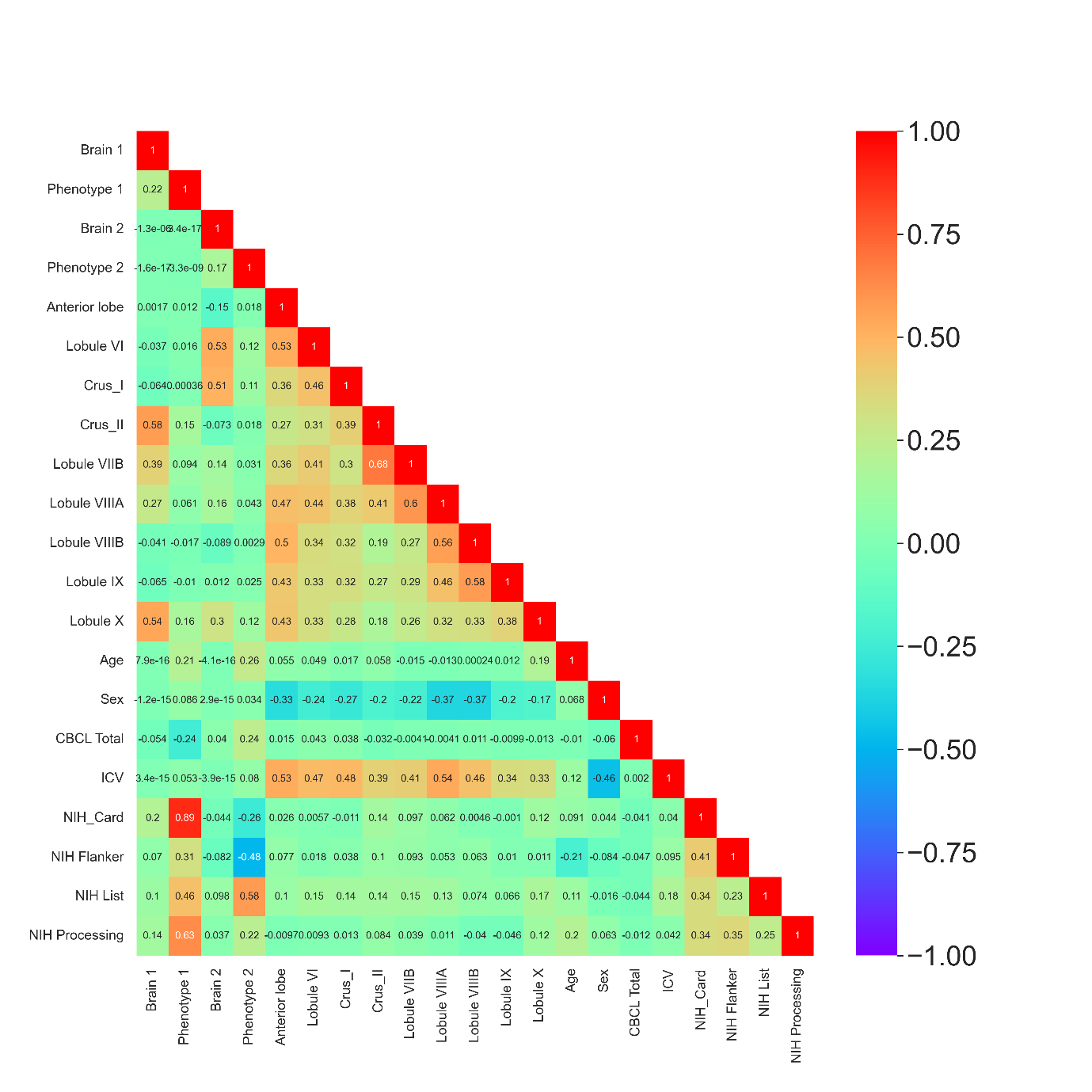


**Figure S3.** Correlation matrix of the CCA results of the relationship of cerebellar structure with cognition and psychopathology. *Annotations*: Brain 1 = first anatomical component; Brain 2 = second anatomical component; Cognition 1 = first cognitive component; Cognition 2 = second cognitive component; NIH Toolbox = NIH-TB; NIH List=NIH TB List Sorting Working Memory Test; NIH Card= NIH TB Cognition Domain Dimensional Change Card Sort Test; NIH Flanker = NIH TB Flanker Inhibitory Control and Attention Test; NIH Processing = NIH-TB Pattern Comparison Processing Speed Test; ICV=Intracranial Volume; CBCL total= Child Behavior Checklist total t-score.

**
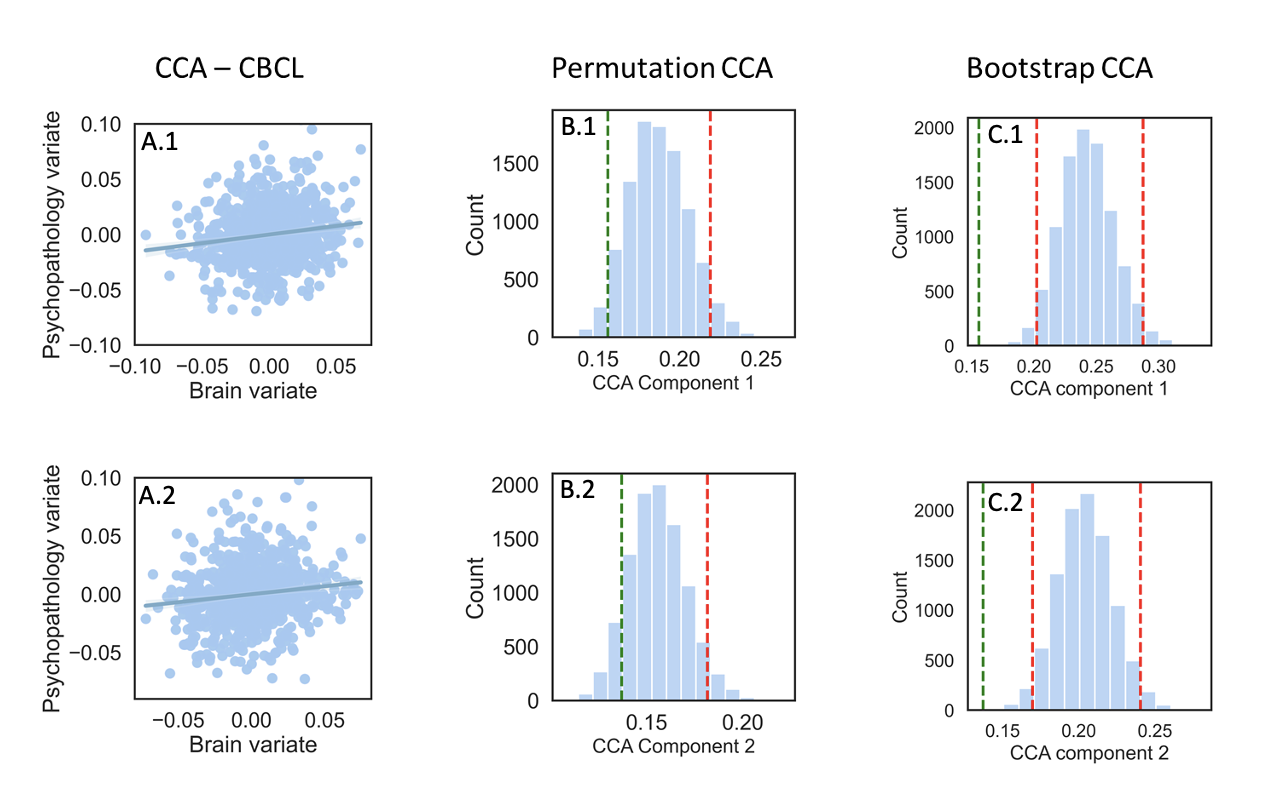
**

**Figure S4. Results of the CCA with CBCL subscores.** A. Canonical correlation plots between the cerebellar and clinical variates for the first component (top panel) and the second (bottom panel) B. Significance testing of the CCA. Distribution of CCA coefficients for component 1 (top) and component 2 (bottom) obtained by performing 10’000 permutations. Red line represents a significance threshold set for an alpha level of 0.05. C. Stability testing of the CCA. Distribution of canonical correlation coefficients between cerebellar and clinical variates by bootstrapping procedure with 10 '000 tests at an alpha level of 0.05 for component 1 (top) and component 2 (bottom). Lower and upper bound corresponding to +/- 1.96 SD in red dotted line
